# Cognitive Restructuring during Depressive Symptoms: A Scoping Review

**DOI:** 10.3390/healthcare12131292

**Published:** 2024-06-28

**Authors:** Bruno Santos, Lara Pinho, Maria José Nogueira, Regina Pires, Carlos Sequeira, Pilar Montesó-Curto

**Affiliations:** 1 Department of Nursing, ESSCVP Alto Tâmega, 5400-673 Chaves, Portugal; 2 Department of Nursing, University of Rovira i Virgili, 43003 Tarragona, Spain; mariapilar.monteso@urv.cat; 3 Department of Nursing, University of Évora, 7000-811 Évora, Portugal; lmgp@uevora.pt (L.P.); maria.nogueira@uevora.pt (M.J.N.); 4 Comprehensive Health Research Centre (CHRC), University of Évora, 7000-811 Évora, Portugal; 5 Nursing School of Porto, CINTESIS of University of Porto, 4200-072 Porto, Portugal; regina@esenf.pt (R.P.); carlossequeira@esenf.pt (C.S.)

**Keywords:** cognitive restructuring, depression, depressive symptoms, nursing, psychotherapy, cognitive behavioral therapy

## Abstract

Cognitive restructuring (CR) aims to get people to challenge and modify their cognitive distortions, generating alternative, more adaptive thoughts. Behavioral, emotional, and physiological responses are modified by analyzing and changing dysfunctional thoughts. The person must have the cognitive capacity to participate in the analysis of their thoughts. CR for people with depression has positive effects, although there is little research on how it should be structured and applied. CR is a thought modification technique presented in the Nursing Interventions Classification (NIC), but is not organized in a sequential approach, and there is no procedure for applying it in practice. This scoping review aims to identify the structure, contents and assessment instruments used in CR for people with depressive symptoms and to analyze the health outcomes of applying the CR technique in this population. Out of 515 articles, seven studies were included in the review, up to 2021 and without any time limitation. The studies were not guided by a consistent and sound framework of the CR technique and each study used its own framework, although they used similar techniques. We grouped CR into six steps. No specific studies were found regarding intervention by nurses. CR is effective in reducing depressive symptoms, so it is an important therapeutic tool that should be used on people with depression. With this scoping review, mental health nurses will have a more comprehensive idea of the techniques that can be used in the application of CR to patients with depressive symptoms.

## 1. Introduction

Depression impacts approximately 300 to 350 million people worldwide. It is a condition that affects a person’s functioning, limiting the person’s autonomy and their role in the family and community. Since depression can appear when a person is still young, it can affect more years of their life, having a high personal and social impact [1,2].

The total number of people estimated to be living with depression increased by 18.4% between 2005 and 2015. The proportion of the global population with depression in 2015 was estimated at 4.4 percent. Depression proved to be more common among women (5.1 percent) than men (3.6 percent). This disorder is widely prevalent in the human population, impacting mood and feelings. It represents a pathological condition, and it is distinct from certain feelings such as sadness, stress, or fear that one may experience at a certain period in life [1].

In consequence of the COVID-19 pandemic, the impact of depression is likely to worsen in the human population [3].

Depressive disorders are characterized by sadness, loss of interest or pleasure, feelings of guilt or low self-esteem, changes in sleep or appetite, feelings of tiredness, and poor concentration. They include two main subcategories: major depressive disorder/depressive episode, classified as mild, moderate, or severe; and dysthymia, which presents as a persistent or chronic form of mild depression. The episodes of dysthymia are similar to a depressive episode but tend to be less intense and last longer [4].

Depression can be long-lasting or recurrent, substantially impairing a person’s ability to function at work or academically, or to cope with their day-to-day life. In its most severe presentation, depression can lead to suicide [1,4,5].

Given the data presented, it is understood that it is necessary to adopt valid strategies in reducing the incidence and impact of this disease in the population.

For mild-to-moderate depression, psychological intervention such as cognitive therapy (CT) may be recommended as a first line of treatment [6]. CT is also especially recommended, as one of the first line treatments, in the treatment of depression during pregnancy and breastfeeding by women. In this group, antidepressant medication should be avoided as much as possible [6,7,8,9]. This type of therapy can be used alone, in the treatment of mild-to-moderate depressive disorders, or combined with antidepressant medications for the treatment of major depression [8].

From this perspective of cognitivist theories, the human organism responds primarily to cognitive representations of its environment and not directly to the environment itself. These cognitive representations are related to learning processes, which are mostly cognitively mediated. Thoughts, feelings, and behaviors are observed to be causally interactive. Behaviors and emotions are related to thoughts that sustain and modulate them [8].

Cognitive therapy was developed by Aaron Beck in the 1960s, who developed a theory of depression centered on cognitive aspects, in which symptoms were related to a negative profile of thinking in three domains: self, world, and future. This theory became known as Beck’s cognitive triad. In his theory, the etiology of psychological disorders is found in the way one conceptualizes reality, with emotional disorders being the consequences of maladaptive thoughts. The author defended the existence of automatic thoughts (cognitions that arise quickly to situations that we experience and remember or project, and that are usually not subject to a more structured rational analysis) and cognitive schemas (set of ideas that reflect how the person organizes information about himself and the surrounding environment) [7,8].

Other authors also contributed to the development of cognitive therapy, such as Kelly, with the Theory of Personal Constructs (core beliefs or self-schemas), which appeared in 1955, based on the understanding of the complexity of others and oneself [7]. Similarly, Albert Ellis, with rational emotive therapy, which emerged in the 1950s, assumed that thoughts, emotions, and behaviors are always interconnected. This author argues that emotions and behaviors result, not from the events themselves, but from how people interpret them [10].

One of the cognitive intervention techniques is cognitive restructuring. Cognitive restructuring (CR) aims to identify negative and unrealistic interpretations (cognitive distortions/dysfunctional thinking) of an event and replace them with more realistic interpretations [11]. Like most CT techniques, it assumes that perception and experience are active processes of internal and external cognitive analysis [7,8]. This intervention aims to modify the behavioral response, changing automatic thinking and consequent emotions, creating alternative behavioral responses. It aims to lead the person to question the validity of their beliefs, analyzing, and modifying them [8,12,13,14].

Throughout their personal and cognitive development, people form brain connections and core beliefs that regulate their attitude and conduct. These beliefs are behind the thoughts that arise in specific situations, such as automatic thoughts. These are interconnected with behaviour in each situation and with the emotional experience. Dysfunctional core beliefs lead to dysfunctional automatic thoughts and maladaptive behaviours, which can lead to the onset of depressive symptoms. With the help of a professional, CR aims to help people change their dysfunctional beliefs and thoughts to a more appropriate form and consequently reduce their depressive symptoms [7,12,14].

Cognitive restructuring is the most essential and theoretically the main mechanism of change [15]. CR presents itself as a useful intervention because of its practical and objective applicability, having concrete results in developing skills to cope with negative emotions and generate more adaptive responses [16,17].

The purpose of CR is based on the relief of the person’s psychological suffering by changing the way they think, as well as the way they interpret and reflect about their experiences, helping users to assess their cognitions not as indisputable facts, but as hypotheses to be tested against logical evidence [7,8,18]. CR is defined in the Nursing Interventions Classification (NIC), with code 4700, meaning a “challenge to the patient to alter distorted patterns of thinking and perceive themselves and the world more realistically”. Although a set of nursing activities corresponding to CR are defined in the NIC, they are not organized in a sequential approach, and there is no procedure for applying CR in practice [19].

Taking into account the above, the need arose to conduct detailed research on the application of CR to people with depressive symptoms to analyze the contents and the procedures used in the application of this therapy, and for the interest and usefulness it has in practice as a specialized intervention in mental health nursing. For this purpose, a scoping review (ScR) on the CR applied to people with depressive symptoms was conducted.

The main aims of this review were to identify the structure, contents, and assessment instruments used in cognitive restructuring for people with depressive symptoms and to analyze the health outcomes of applying the cognitive restructuring technique to people with depressive symptoms.

## 2. Materials and Methods

Based on the updated scoping review guidance of Pollock et al. [20], this scoping review followed the JBI ScR framework and the PRISMA-ScR extension. The following steps were followed: (1) identify the research questions, (2) identify pertinent studies, (3) select the studies, (4) map the data obtained, (5) gather, summarize, and report the data [21,22].

The scoping review protocol was registered with the Open Science Framework: https://osf.io/jyqsv (accessed on 13 December 2020).

As recommended in the guidelines for scoping reviews, we formulated the research questions based on the ‘PCC’ format: Population, Concept, and Context [23].

Question 1: What are the characteristics (structure, contents, and assessment instruments) of cognitive restructuring used in people with depressive symptoms?

- Population: people with depressive symptoms,
- Concept: the structure, contents and assessment instruments of cognitive restructuring,
- Context: all clinical settings where the target population undergoes cognitive restructuring.

Question 2: What are the health outcomes of applying the cognitive restructuring technique to people with depressive symptoms?

- Population: people with depressive symptoms,
- Concept: the health outcomes of applying the cognitive restructuring technique,
- Context: all clinical settings where the target population undergoes cognitive restructuring.

### 2.1. Inclusion and Exclusion Criteria

The criterion for inclusion were studies on cognitive restructuring (CR) applied to people with depressive symptoms, which included the structure and content of the intervention. Initially, we searched for primary and secondary studies on the subject in a 10-year timeframe. Due to the small amount of specific information available on the subject under study, we changed the search to an open-ended one to map the largest amount of information.

The languages defined as criteria were English, Portuguese, and Spanish, due to the vast amount of information they allow us to map. These languages are the ones most fluently mastered by the reviewers in the study. This ensured that the selection and analysis of the ScR process had the utmost rigor.

Studies involving children were excluded, due to the specificity of the interventions in this age group. Psychotherapeutic interventions with children differ in structure and content, because children are at a very different stage of cognitive development from the adult population. It was decided not to exclude the adolescent age group, due to the similarity of some interventions, in original or adapted form, used in both adults and adolescents. Adolescents have a more developed understanding of cognitive processes than children.

By adolescent we mean a person between the ages of 13 and 18, and by adult we mean a person over the age of 18 [24].

### 2.2. Search Strategy

To minimize bias in the ScR results, the screening and data extraction process was performed by two independent reviewers and a third reviewer to analyze discrepancies.

The databases included MEDLINE with full text (via EBSCOhost); CINAHL plus with full text (via EBSCOhost); Psychology and Behavioral Sciences Collection (via EBSCOhost); Academic Search Complete (via EBSCOhost); SPORTDiscus with full text (via EBSCOhost); RCAAP; ARROW; Australian National University-open research; and OpenGrey.

The methodological strategy of the search, to include information from the grey literature, was due to the existence of various types of documents of practical and scientific interest (at the private or organizational level), increasing the relevant information to map [21].

The database search was performed in April 2021.

With support from the selected databases, the keywords were determined based on vocabulary terms from the medical subject heading (MeSH), which best translated the focus of the search to allow a consistent means of searching for the information [24]. The following terms were identified: “cognitive restructuring”, “depression”, and “depressive symptoms”.

The term cognitive restructuring does not appear as an exact term in the descriptors in DeSC or MeSH, being, however, a relevant term for our search. It was thus considered relevant by the researchers to include this term, allowing for more precise information to be sought. We also agree that the inclusion of this term in the Boolean phrase would not limit the search in scientific databases, due to the way it is constructed.

The search was performed with the following Boolean phrase: (“cognitive restructuring” OR “cognitive therapy”) AND (“depression” OR “depressive symptoms”) NOT “child”.

All types of studies found were included, with the rationale that the ScR should be based on data from any type of evidence and research methodology and is not restricted to only quantitative studies (or any other study design) [25].

### 2.3. Study Selection and Extraction

Data selection and extraction was done based on the template instrument presented by JBI for extracting details, characteristics, and outcomes [25]. These instruments were adapted based also on the objectives of the review. The title and abstract were read, according to the previously established search and inclusion criteria. A full text analysis of the articles was performed on those that met the selected criteria as described in the study protocol.

Articles in which both reviewers agreed on their interest for the research were included. Whenever there was no agreement between the reviewers, a discussion with a third reviewer was used. From the selected articles, an analysis of the reference lists was performed, first by the title, followed by an analysis of the abstracts considered relevant, and then by an analysis of the full text. Additional relevant articles that were not duplicated were included in the ScR.

In the search, after removing duplicate records, 515 records were found. After the screening process, 60 articles were selected for full-text analysis. By the eligibility criteria, 7 articles were selected for inclusion in the ScR.

**Figure 1:**
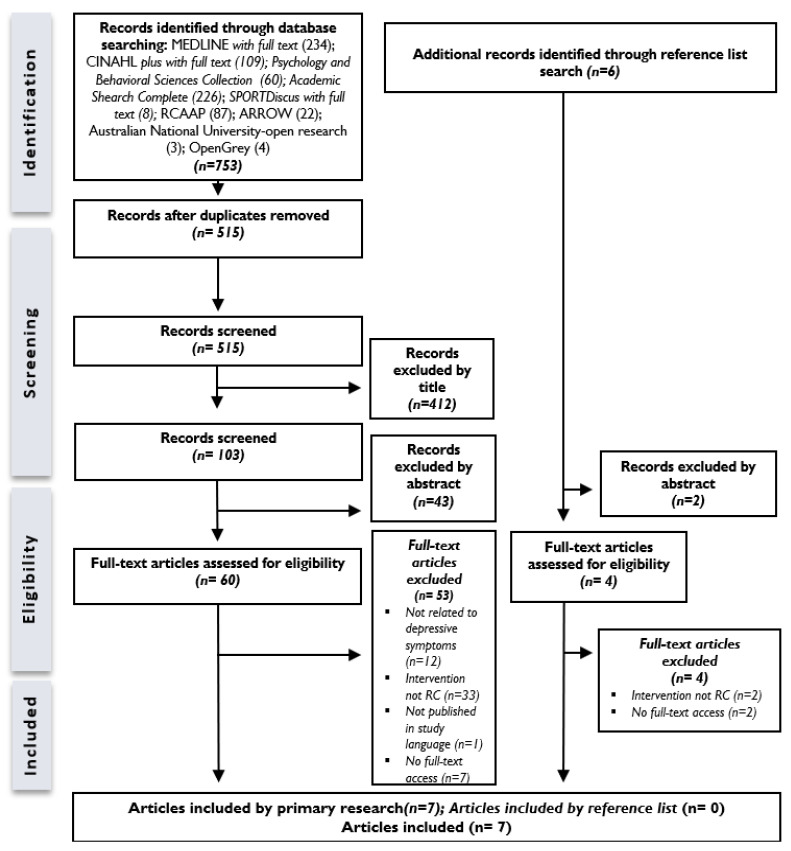
PRISMA flow chart demonstrating search strategy.

The information search and selection process are summarized in Figure 1—PRISMA, a flow chart demonstrating search the strategy.

## 3. Results

According to the mapped studies, CR sessions range from micro-interventions of one session [26,27] to three sessions [28], longer interventions of eight sessions [29], and nine to twelve sessions [30] or sixteen sessions [31]. Shorter interventions were conducted on consecutive days [28] and longer interventions were conducted on a weekly [31] or bi-weekly basis [29]. The duration of each session ranged from 60 min [29] to 90 min [12] or 120 min [30]. Only one of the studies performed a follow up, which took place after 6 months [32]. In all studies, the reference technician was the psychologist.

One exploratory study aimed to identify the evolution of cognitive restructuring techniques, with results indicating that in the most successful cases of CR, a greater number and frequency of techniques are used [31].

Three articles are experimental studies, with two of these studies demonstrating the usefulness of CR in reducing depressive symptoms [29,32], and one finding that a combination of emotional processing (EP) and CR was the most effective intervention, compared with the single use of each of these two interventions [28].

One study is a pilot study with a sample of female cancer patients and proved the effectiveness of CR in reducing depressive symptoms [27].

Another two articles are randomized controlled trials (RCT), which also obtained therapeutic improvements with CR in people with depressive symptoms [26,30]. Table 1 describes a summary of the included articles.

The assessment instruments used in the studies are shown in Table 2. The Beck Depression Inventory-II (BDI-II) was the most used instrument to assess depressive symptoms [26,27,28,30,31], with the remaining instruments not repeated in any of the other studies.

Table 3 shows the structure and techniques used in the studies when using CR. For better structuring and organization of the sessions we grouped the techniques into the following steps: Start; Explore; Identifying; Comprehension; Changing Beliefs; and Finalizing. We also present conducts that should be present in all sessions.

**Figure 2:**
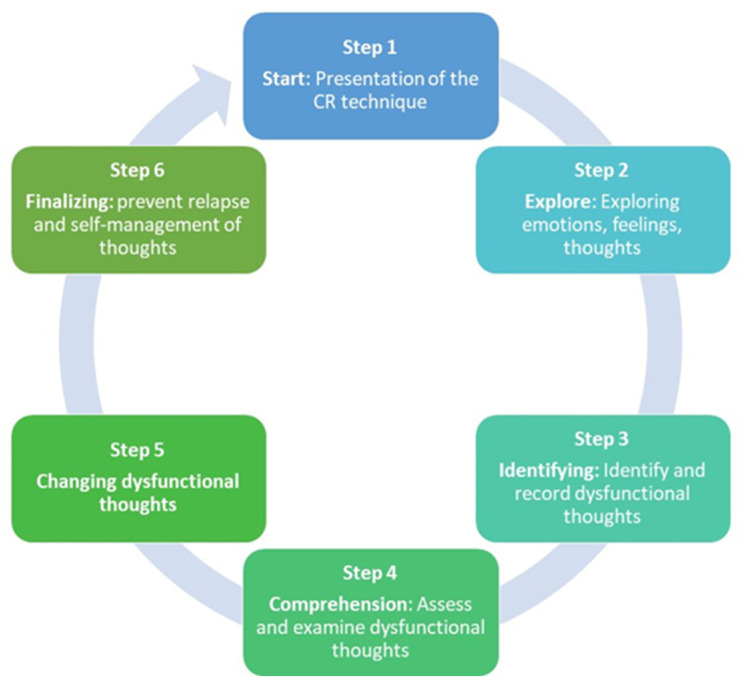
Cognitive Restructuring Structure.

The steps shown in Table 3 are summarized in Figure 2. This figure shows the structure that emerges from the results analyzed in the studies included in the ScR.

## 4. Discussion

The results of applying CR demonstrate a reduction in depressive symptoms [26,27,28,29,31,33]. In addition to reducing depressive symptoms, CR has also been shown to be effective in improving self-esteem and reducing stress levels [29]. One study indicates that clinical improvement usually comes after the 6th–8th session [34]. However, one study indicates that even with a single session, positive effects can already be detected [32]. CR is an important non-pharmacological intervention for people with depression.

The application of CR requires a complete mastery of the focus of intervention, as well as dynamic skills and therapeutic creativity, in order to maintain the fluidity of the sessions and a relationship of trust with the person assisted. Given the diversity of techniques applied in CR, the therapist must have a high level of mastery of them. Results indicate that in the most successful cases of CR, a greater number and frequency of techniques are used [31]. The number of strategies increases in frequency between the 1st and 8th sessions and, consequently, in the last sessions (12th and 16th). In these sessions, the number of strategies is no longer so evident, since the process is being completed and the therapist no longer needs to frequently use the different strategies [31].

The diversity and flexibility in the application of CR is related to the need to adapt the techniques to each person [13], although the results of this scoping review also point to a standard structure and goals defined in the sequence of sessions. The sessions are linked by a logical sequence, which allows cognitive and behavioral modifications to be integrated. CR also appears to have a greater number of conventional presentations [35]. More structured and objective psychotherapeutic interventions bring very satisfactory and shorter-term results than longer and subjective interventions [36]. However, in some situations, the CR intervention may require a larger number of sessions than usual [37]. The number and duration of the CR sessions was variable, which shows flexibility in its applicability, with CR seeming to move away from more rigid and lengthy models. Decreased cognitive flexibility and executive ability can lead to difficulty integrating CR and achieving positive results [38]. Thus, in addition to the importance of structuring the CR technique, it is also important to carry out an individual cognitive conceptualization treatment plan [7]. A recent integrative literature review concluded that care plans for people with depression should be individualized, dynamic, flexible, and participatory [39], which is in line with this perspective.

Psychotherapeutic intervention, including CR, should focus on adapting the techniques to each person, their internal processes, and personal experiences. The need to establish an empathic and trusting relationship is emphasized to achieve success in CR [40].

Through analysis of the various articles in this scoping review, we were able to group CR by steps, the first step being the presentation of the therapist and participant and an explanation of the intervention, as well as its goals. This first step is fundamental for the beginning of the relationship and the success of the intervention and will support step 2, which is the exploration phase where the problem is explored, being crucial for the catharsis and the identification of emotions, feelings, and thoughts. If we think of Peplau’s theory [41], we can make a link and say that these first two steps correspond to the orientation phase. Step 3 is the identification and recording of dysfunctional thoughts (automatic thoughts, rumination, negative thoughts, irrational beliefs, etc.). We can say that it would correspond to the identification phase of Peplau’s theory. In step 4, it is intended that there is an understanding of dysfunctional thoughts through their evaluation and analysis, making the person aware of cognitive errors. This step could correspond to the exploration phase of Peplau’s theory. In step 5, the goal is to change the beliefs and cognitive errors, that is, through the analysis performed in step 4, it is intended that the person becomes aware of their thoughts and dysfunctional beliefs and changes attitudes and behaviors. The completion phase is intended to prepare the person to prevent relapse and to self-manage their dysfunctional thoughts in their daily life. Steps 5 and 6 would correspond to the resolution phase of Peplau’s theory. 

The need to establish a structure for psychotherapeutic interventions, such as CR, is highlighted by some authors [42,43].

For the assessment of CR outcomes in relation to depressive symptoms, the Beck Depression Inventory-II (BDI-II) was the most commonly used instrument [26,27,28,30,31]. However, other authors have used the Center for Epidemiological Studies Depression Scale (CES-D) [32], the Depression Anxiety Stress Scales (DASS) and the Geriatric Depression Scale of Yesavage (GDS) [29]. Instruments were also used to assess Self-Esteem [29], Resilient level [29], Subjective discomfort; Interpersonal relationships; Social role performance [31], Social support, Cognitive Symptoms, Basic activities of daily life [29], Satisfaction with Life [28,29] and Pet-Related Distress [28].

In the Nursing Interventions Classification (NIC) manual, cognitive restructuring emerges as a relevant technique in nursing care that supports psychosocial functioning and facilitates life changes [19]. However, no studies emerged in our research in which the therapist was a nurse. These results indicate the need for a greater focus on evidence-based psychotherapeutic interventions by specialist nurses in mental health and psychiatry, as well as the dissemination of research studies. This scoping review aims to be a first step towards the increased use of this type of interventions by specialist nurses, as a basis for the application of CR to people with depression.

According to the Psychotherapeutic Intervention Model in Nursing, cognitive restructuring can be used as a psychotherapeutic intervention by the nurse specialist in mental health and psychiatric nursing, always based on a nursing diagnosis [42,43]. There are several nursing diagnoses in which the psychotherapeutic intervention of cognitive restructuring can be used, but its use requires specialized knowledge in this diagnostic assessment.

There are very few research studies that have used the cognitive restructuring technique with people with depression and we have only found two RCTs. We did not find any studies where the therapist was a mental health nurse specialist. This ScR reflects the need for primary research into CR carried out by mental health nurses on people with depressive symptoms.

## 5. Conclusions

CR is effective in reducing depressive symptoms, improving self-esteem, and reducing stress levels, so it is an important therapeutic tool that should be used on people with depression. The few research studies that have applied CR suggest that there is a need for greater investment in this type of non-pharmacological intervention to help people manage their dysfunctional thoughts and thereby reduce depressive symptoms and disability.

Although the information on CR was scattered and not standardized, it was possible to map and characterize the intervention. The studies were not guided by a consistent and sound framework of the CR technique and each study used its own framework, although they used similar techniques. This suggests the need for a structured manual to support the interventions to make their application more rigorous and less subjective. It is our intention to continue researching this subject in order to draw up a support manual with different steps and strategies to help implement CR. In practice, a manual will help provide specific training on the CR technique. The different stages, structure, and content of CR resulting from this research could contribute to define further steps of more advanced research. We emphasize, however, the importance of adapting the techniques to each person’s individuality and needs. The existence of a manual of RC, does not eliminate the need for personalized adjustments to meet people’s specific needs, ensuring a more effective and person-centered approach. Although CR is documented in the NIC, there is no structured CR intervention for nursing intervention based on focus nursing. Besides that, no specific studies were found referring to the intervention of nurses. This suggests the need to group and structure the data obtained for the construction of a psychotherapeutic intervention in CR nursing through consultation with mental health nursing experts so that nurses can then use evidence-based CR. This ScR was the first step in a broader investigation that we intend to carry out, namely the conduct of a focus group, a pilot study, and a randomized controlled trial, to obtain a scientifically validated and structured CR intervention aimed at differentiated intervention for mental health nurses. The identification of seven records for analysis in the ScR also suggests the need for primary research into CR in people with depressive symptoms.

## Figures and Tables

**Table 1:** Studies, aims and main results.

Authors	Study	Aim	Results
(Araújo and Santos, 2015)	Exploratory	To identify the evolution of cognitive restructuring techniques.	(…) in the unsuccessful case, the therapist did not have the opportunity to deepen the CR techniques for modifying intermediate and core beliefs (…). (…) cognitive restructuring techniques, in the successful cases, decrease over time, in contrast to the unsuccessful cases that decrease until the intermediate phase, but increase again in the final phase. It is noteworthy that not only the use of techniques varies, but also their quantity (…) (…) the symptomatology only decreased, although not significantly (…) where the CR techniques were used more frequently.
(Hunt et al., 2007)	Experimental	To compare the effects of CR and emotional processing (EP) following a depressing life event.	The combination of EP and CR was the most effective intervention (…). One month later, participants in the combined condition showed the greatest recovery from depressive symptoms, followed closely by those in the EP alone. The EP and EPCR groups did not differ significantly in outcome on the BDI (…) and this pattern of results suggests that habituation was not the sole mechanism for improvement in depressive symptoms.
(Asuzu, et al., 2015)	Pilot Study	To present the feasibility and acceptability of a pilot cognitive restructuring intervention and its impact on depression outcomes in a group of female cancer patients in Nigeria.	Female cancer patients exposed to cognitive restructuring experienced reduced depression compared to their baseline depression scores.
(Aderka, et al., 2013)	RCT	To examine relationship between post-traumatic and depressive symptoms during prolonged exposure (PE) treatment with and without CR.	This pattern of results suggests that PE primarily affects post-traumatic symptoms, which in turn affect depressive symptoms. PE/CR results in a more reciprocal relationship between post-traumatic and depressive symptoms.
(Deldin and Chiu, 2005)	RCT	To examine whether baseline patterns and levels of cortical activity may be useful indices of mood response to a cognitive therapy.	CR decreased depressive symptoms and led to EEG changes.
(Sales et al., 2015)	Experimental	To prove that the application of cognitive restructuring therapy in depressed older adults is effective for the improvement of two symptoms.	CR in older adults has been satisfactory, showing the usefulness of this type of therapy. Cognitive restructuring has been shown to be effective in improving self-esteem and significantly reducing stress levels.
(Zaunmüller et al., 2014)	Experimental	To investigate the affective impact and electrocortical correlates of cognitive restructuring.	Positive effects of CR were revealed, even with a single session.

**Table 2:** Assessment Instrument.

Indicator	Assessment Instrument	Study
Depressive symptoms and severity of depression	Beck Depression Inventory-II (BDI-II)	Araújo and Santos, 2015 Hunt et al., 2007 Asuzu et al., 2015 Deldin and Chiu, 2005 Aderka et al., 2013
	CES-D, Center for Epidemiological Studies Depression Scale	Zaunmüller, et al., 2014
	Depression Anxiety Stress Scales (DASS)	Sales et al., 2005
	Geriatric Depression Scale of Yesavage (GDS)	Sales et al., 2005
Self-esteem	Rosenberg Self-Esteem Scale	Sales et al., 2005
Resilience level	Brief Resilient Coping Scale	Sales et al., 2005
Subjective discomfort; interpersonal relationships; and social role performance	Outcome questionnaire (OQ-45)	Araújo and Santos, 2015
Social support	MOS Social Support Survey	Sales et al., 2005
Cognitive symptoms	Mini Examen Cognoscitivo (MEC)	Sales et al., 2005
Basic activities of daily life	Barthel Index	Sales et al., 2005
Satisfaction with life	Satisfaction with Life Scale (SWLS)	Hunt et al., 2007
	The Philadelphia Geriatric Center Morale Scale	Sales et al., 2005
Coping	Ways of Responding (WOR) Rater’s Scale	Hunt et al., 2007
Pet-related distress	Specific Pet-Related Distress (EMO)	Hunt et al., 2007

**Table 3:** CR Structure and Techniques.

Structure	Techniques	Study
Start	Presentation of the program, objectives, number of sessions and approximate duration	Sales et al., 2015
	Introduce the therapist and the participants in order to create a climate of trust and security	
	Explanation of the Cognitive Theory of Depression	
	Brief explanation of CR, thoughts and influence on behavior, set examples.	Araújo and Santos, 2015; Aderka et al., 2013; Deldin and Chiu, 2005
Explore	Report of experiences, emotions, and beliefs	Asuzu et al., 2015
	Case conceptualization	Araújo and Santos, 2015
	Brainstorming	Sales et al., 2015
	Technique: evoke automatic thoughts	Araújo and Santos, 2015
	Socratic method	Aderka et al., 2013
	Homework: recording of thoughts and emotions (diary).	Araújo and Santos, 2015; Asuzu et al., 2015; Aderka et al., 2013
Identifying	Identifying dysfunctional thoughts	Hunt et al., 2007; Deldin and Chiu, 2005; Zaunmüller et al., 2014
	Descending arrow technique	Araújo and Santos, 2015
	Identification of irrational beliefs (intermediate and central)	Sales et al., 2015; Araújo and Santos, 2015
	Identifying ruminant thoughts	Asuzu et al., 2015
	Automatic thought identification technique	Araújo and Santos, 2015
	Registration of distorted thoughts and irrational beliefs	Sales et al., 2015; Aderka et al., 2013
	Recording the frequency of negative thoughts	Hunt et al., 2007; Deldin and Chiu, 2005
	Elaboration of the list of problems	Sales et al., 2015
	Identify and target whatever beliefs help feel better	Hunt et al., 2007
Comprehension	Assess and examine the validity of automatic thought, challenge and debate maladaptive thought	Araújo and Santos, 2015, Asuzu et al., 2015
	Exploring the possibility of other interpretations for events and beliefs/reframe thinking	Hunt et al. 2007; Asuzu et al., 2015
	Examining the facts by making a list of the ways negative thoughts could be false	Deldin e Chiu, 2005
	Taught to deal with feelings of guilt, self-blame, and self-inadequacy	Asuzu; Akin-Odanye; Philip, 2015
	Review of how you saw and dealt with past negative events and experiences	Araújo and Santos, 2015; Deldin and Chiu, 2005; Sales et al., 2015
Changing dysfunctional thoughts	Developing alternative explanations for negative situations and thoughts (Strategies More Adaptive)	Araújo and Santos, 2015; Deldin e Chiu (2005), Sales et al., 2015; Zaunmüller et al., 2014; Hunt et al., 2007
	Modification of intermediate beliefs	Araújo and Santos, 2015
	Core-belief modification technique: developing new core beliefs and strengthening new core beliefs	Araújo and Santos, 2015; Sales et al., 2015
	Modification of dysfunctional thoughts	Deldin and Chiu, 2005
	Technique: taking action to solve the problem	Araújo and Santos, 2015
	Technique: developing new adaptive patterns and assumptions	Araújo and Santos, 2015; Sales et al., 2015
	Replacement of old thinking with new thinking	Sales et al., 2015
	Looking for or finding a positive feature in a seemingly negative situation	Hunt et al., 2007; Zaunmüller et al., 2014
	Positive self-statements: the negative thoughts and self-talks by replacing them with positive and empowering ones	Asuzu et al., 2015
	Restructuring skills were reinforced and practiced by applying them to two film clips (10 min excerpts from movies)	Zaunmüller et al., 2014
	Self-realized prophecies	Araújo and Santos, 2015
	Imaginal exposure	Aderka et al., 2013
	Breathing exercise with guided imagery	Asuzu et al., 2015
	Diminishing the importance of a negative situation	Hunt et al., 2007
	Substituting neutral phrases for emotionally loaded words	Deldin and Chiu, 2005
Finalizing	Taught how to prevent relapse and how to use the skills learnt in self-management	Asuzu et al., 2015; Aderka et al., 2012; Sales et al., 2015; Zaunmüller et al., 2014
	Plan the continuation of exercises learned during the CR sessions	Sales et al., 2015
All Sessions	Began with homework review and ended with homework assignment.	Aderka et al., 2013
	Brief descriptions of the treatments follow.	Aderka et al., 2013
	To clarify doubts	Sales et al., 2015
	Summarized what they had learned in treatment and discussed their progress.	Aderka et al., 2013, Sales et al., 2015
	Reflection on previous sessions	Hunt et al., 2007

## Data Availability

Not applicable.
